# *Mycobacterium ulcerans* in Mosquitoes Captured during Outbreak of Buruli Ulcer, Southeastern Australia

**DOI:** 10.3201/eid1311.061369

**Published:** 2007-11

**Authors:** Paul D.R. Johnson, Joseph Azuolas, Caroline J. Lavender, Elwyn Wishart, Timothy P. Stinear, John A. Hayman, Lynne Brown, Grant A. Jenkin, Janet A.M. Fyfe

**Affiliations:** * Austin Health, Melbourne, Victoria, Australia; † University of Melbourne, Melbourne, Victoria, Australia; ‡ Victorian Infectious Diseases Reference Laboratory, North Melbourne, Victoria, Australia; § Monash University, Melbourne, Victoria, Australia; ¶ Department of Primary Industries, Melbourne, Victoria, Australia; # Department of Human Services, Melbourne, Victoria, Australia

**Keywords:** Mycobacterium ulcerans, Buruli ulcer, Bairnsdale ulcer, mosquito, vector, transmission, Australia, research

## Abstract

Buruli ulcer (BU) occurs in >30 countries. The causative organism, *Mycobacterium ulcerans*, is acquired from the environment, but the exact mode of transmission is unknown. We investigated an outbreak of BU in a small coastal town in southeastern Australia and screened by PCR mosquitoes caught there. All cases of BU were confirmed by culture or PCR. Mosquitoes were trapped in multiple locations during a 26-month period. BU developed in 48 residents of Point Lonsdale/Queenscliff and 31 visitors from January 2001 through April 2007. We tested 11,504 mosquitoes trapped at Point Lonsdale (predominantly *Aedes camptorhynchus*). Forty-eight pools (5 species) were positive for insertion sequence IS*2404* (maximum likelihood estimate 4.3/1,000), and we confirmed the presence of *M. ulcerans* in a subset of pools by detection of 3 additional PCR targets.

## Abstract 2

Mosquitoes positive for *M*. *ulcerans* were linked to outbreaks of Buruli ulcer in humans.

Buruli ulcer (BU), also known as Bairnsdale ulcer (*1*), Daintree ulcer (*2*), and Mossman ulcer in Australia, is an emerging disease of skin and soft tissue with potential to cause scarring and disability (*3*). It is caused by *Mycobacterium ulcerans* (*4*), an environmental pathogen that produces a destructive polyketide toxin, mycolactone (*5*); the genes for the production of this toxin are encoded on newly described plasmid *p*MUM001 (*6*). BU occurs in >30 countries worldwide, but it affects mainly children in sub-Saharan Africa, where it is now more common than tuberculosis and leprosy in some regions (*7*). This disease occurs in people of all ages and races who live in or visit BU-endemic areas, but the precise mode of transmission remains unknown.

Analysis of the recently sequenced *M*. *ulcerans* genome has shown that in addition to *p*MUM001, there are unusually high copy numbers of 2 independent insertion sequences (IS*2404* and IS*2606*) and a high incidence of pseudogene formation (*8*). These data suggest that *M*. *ulcerans* is unlikely to be free-living in the environment but is instead undergoing adaptation to a specific ecologic niche in which the products of some ancestral genes are no longer essential. One such niche may be in aquatic insects because *M*. *ulcerans* has recently been reported to colonize the salivary glands of carnivorous water bugs (Naucoridae) under laboratory conditions (*9*), and mycolactone production appears to be necessary for this colonization (*10*). Studies from disease-endemic areas in Africa have reported that farming activities near rivers (*11*) and swimming in rivers or marshes (*12*) may be risk factors for BU; bites from contaminated water bugs may transmit the infection.

In temperate southeastern Australia, outbreaks of *M*. *ulcerans* infection occur in localized areas, but few patients report direct contact with environmental water other than the ocean, which led to the proposal that aerosols from contaminated water may cause human infections (*13*). However, these low-lying disease-endemic areas also harbor large populations of mosquitoes, and some patients have reported that BU first appeared at the site of what may have been a mosquito bite (Figure 1). These observations, and knowledge from field studies in Africa implicating insects as either a reservoir or mode of transmission, led us to capture and screen mosquitoes during our investigation of a large outbreak of BU in humans in a small coastal town in southeastern Australia (Point Lonsdale), ≈60 km south of Melbourne (Figure 2).

**Figure 1:**
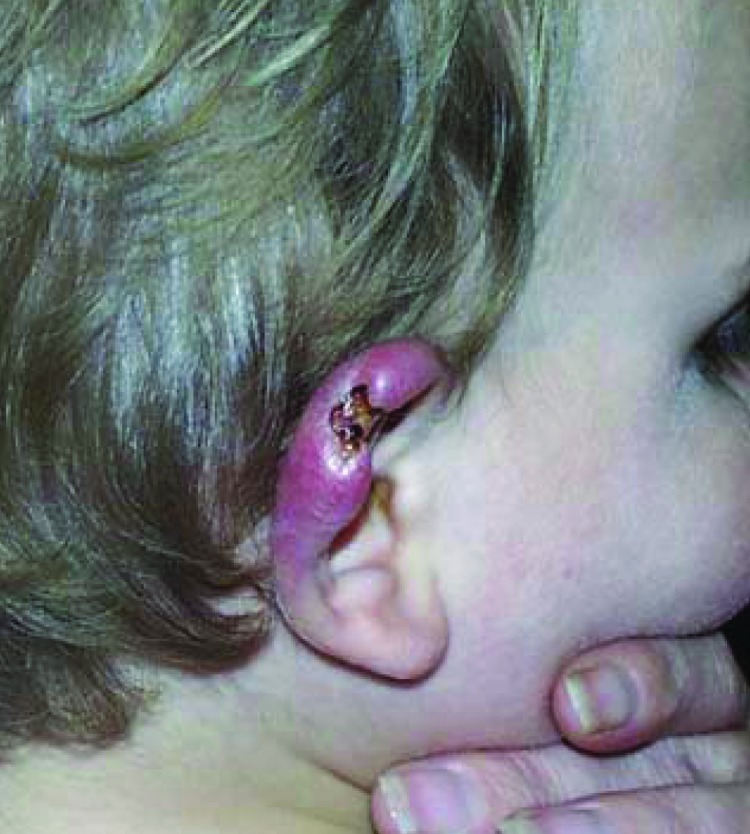
Ear of an 18-month-old child with culture- and PCR-confirmed Buruli ulcer who briefly visited St. Leonards, Australia, in 2001 (Figure 2). The initial lesion resembled a mosquito bite or that of another insect.

**Figure 2:**
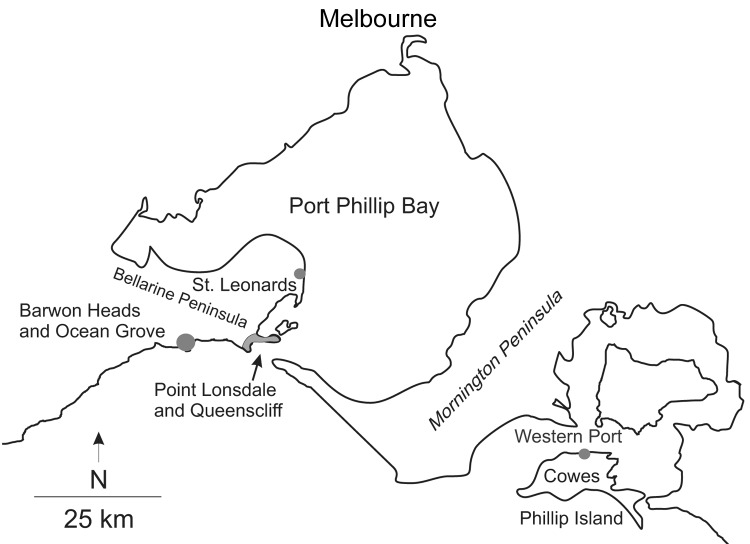
Map of central coastal Victoria, Australia, showing towns and places referred to in the text or in associated references.

## Methods

### Outbreak Investigation

*M*. *ulcerans* infection has become increasingly common in the southern Australian state of Victoria since the early 1990s (*14*,*15*) and characteristically causes localized outbreaks (*16*). In 1995, a research group at the Royal Children’s Hospital in Melbourne developed an IS*2404* PCR to improve speed and accuracy of diagnosis of BU (*17*). This method has now become the initial diagnostic method of choice in Australia and elsewhere (*18*). All PCR- and culture-positive cases of *M*. *ulcerans* infection in Victoria have been unofficially reported to the Victorian Department of Human Services (DHS) since the 1990s, and investigators from DHS began to routinely contact and interview all new reported case-patients in 2000. All new cases of *M*. *ulcerans* infection were made legally reportable in Victoria in January 2004 (*19*).

### Case Definition

For this study, a case of BU was defined as a patient with a suggestive clinical lesion from which *M*. *ulcerans* was identified by PCR or culture from a swab or tissue biopsy specimen from January 2002 through April 2007; the patient must have been either a resident of, or a visitor to, Point Lonsdale or Queenscliff (adjacent coastal towns on the Bellarine Peninsula) who did not report a recent history of contact with another known BU-endemic area. Australian Bureau of Statistics data derived from the 2001 Australian Census for Point Lonsdale/Queenscliff (postcode 3225) were used to obtain the resident population numbers and age distribution in the outbreak area (*20*).

### Mosquito Trapping

A total of 8–13 overnight mosquito traps were placed at Point Lonsdale on 22 occasions from December 2004 through January 2007. Adult mosquito sampling was conducted with CO_2_-baited miniature light traps (*21*). Traps were 2-L, cylindrical, insulated containers designed to hold CO_2_ pellets that continuously produce CO_2_, which then diffuses through holes in the bottom of the container. A small electric light and fan at the base of the container deflected attracted mosquitoes into a holding container. The traps were set before dusk and then retrieved several hours after dawn the next morning. The catches were transported to Primary Industries Research in Attwood, Victoria, where they were counted, sorted, and pooled by sex and species. Mosquito species were identified by using the key of Russell (*22*). All captured mosquitoes were tested except in February–March 2005 and again in October 2005 when recent rains led to large spikes in mosquito numbers.

### Screening of Mosquitoes by PCR

DNA was extracted from pools of <15 individual mosquitoes (occasional pools had <23 mosquitoes) of the same sex and species by using the FastDNA Kit (revision no. 6540-999-1D04) and the FastPrep Instrument (Qbiogene Inc., Irvine, CA, USA) according to the manufacturer’s instructions. We adapted fluorescence-based real-time PCR technology to screen mosquitoes for 3 *M*. *ulcerans*–specific DNA sequences as described (*23*). Briefly, oligonucleotide primers and TaqMan MGB probes (Applied Biosystems, Foster City, CA, USA) labeled with fluorescent dyes 6FAM or VIC were designed that targeted 3 independent high-copy number repetitive sequences (IS*2404* and IS*2606* [*24*] and the ketoreductase B domain [KR] from *p*MUM001 [*6*]). The copy number of these targets per bacterial cell in the published sequenced of *M*. *ulcerans*, to which the outbreak strain is phylogenetically closely related, is 213 for IS*2404*, 91 for IS*2606*, and 30 for KR (*8*). Assays were conducted with an ABI PRISM 7000 Sequence Detection System (Applied Biosystems).

Each pool was first tested for IS*2404* with an internal positive control to test for PCR inhibition and separate negative and positive controls. Samples were considered positive for a given target when they had a result above a previously determined critical threshold (*23*). Pools that were positive for IS*2404* were then screened in duplicate with confirmatory assays to detect IS*2606* and KR. For pools with sufficiently high signal strength, amplification and sequencing of variable number tandem repeat (VNTR) locus 9 were conducted by using a nested PCR. The first round PCR used 2 primers, MUVNTR9NF (5′-ACTGCCCAGACATGGCGA-3′) and MUVNTR9NR (5′-ACGCGAGGTGGAACAAAGC-3′), designed to flank the published VNTR locus 9 primer. First-round PCR products were used as template for a second-round PCR performed as described by Ablordey et al. (*25*). PCR products of the expected size were sequenced by using the BigDye Terminator version 3.1 Cycle Sequencing Kit (Applied Biosystems) according to the manufacturer’s instructions. Precipitated reaction products were tested in a 3730S Genetic Analyzer (Applied Biosystems) (*23*). The maximum likelihood estimate (MLE) per 1,000 mosquitoes tested (bias corrected MLE) was calculated by using software recommended for this purpose by the Centers for Disease Control and Prevention (Atlanta, GA, USA) (*26*).

## Results

### Description of Outbreak

**Figure 3:**
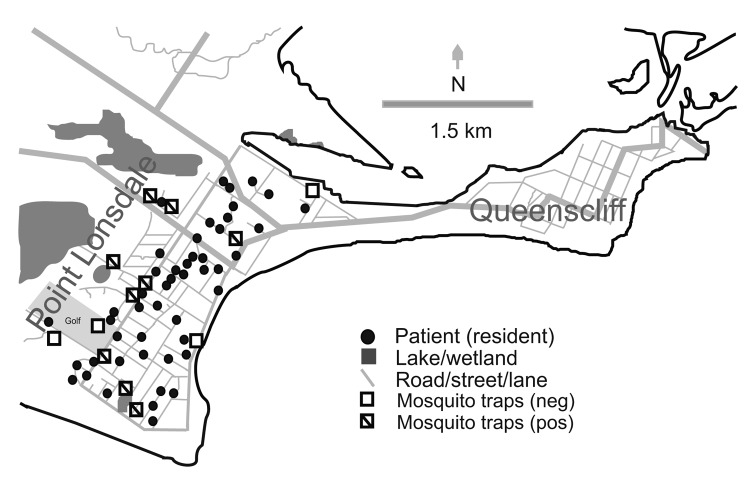
Map of Point Lonsdale/Queenscliff, Australia (postcode 3225), showing location of houses of affected permanent residents, mosquito traps, and other features mentioned in the text. Not all traps yielded PCR-positive mosquitoes during the trapping period. Neg, negative; pos, positive.

The climate in Point Lonsdale is temperate with a mean daily maximum temperature of 12.8°C in July (winter) and 22.4°C in January (summer). Average annual rainfall is 660 mm and is spread throughout the year (e.g., average 41.3 mm in January and 59.1 mm in July) (*27*). Most of the town is low-lying and close to sea level, and there are several natural and human-made swamps and water features in the vicinity (Figure 3). Natural vegetation includes dense clumps of coastal tea trees (*Leptospermum laevigatum*).

Point Lonsdale shares a beach with Queenscliff (Figure 3), a neighboring town 4 km to the east. Point Lonsdale and Queenscliff (postcode 3225) were included in the 2001 Australian Bureau of Statistics Census and had a resident population of 3,851 at that time, but there are also large numbers of visitors to this scenic area during summer holiday periods (e.g., 54,000 people visited the Queenscliff Visitor Information Centre in 2005; pers. comm.).

**Figure 4:**
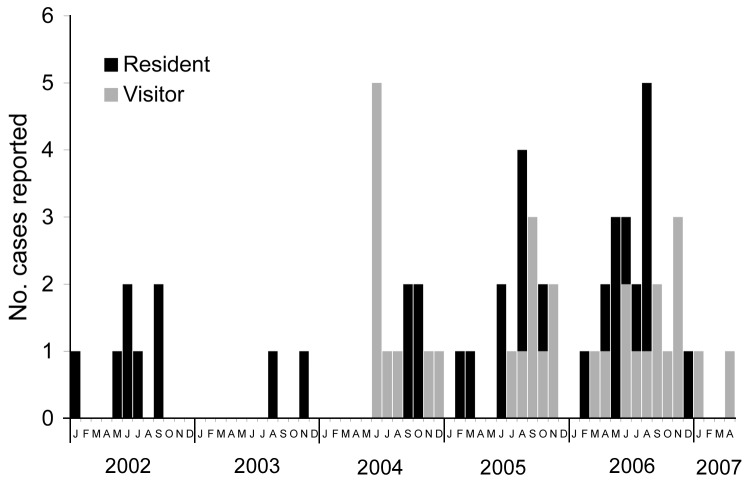
Epidemic curve of cases of Buruli ulcer linked to Point Lonsdale/Queenscliff, Australia, by resident/visitor status and month of reporting, 2002–2007.

*M*. *ulcerans* infection was not found in the area before 2002. From January 2002 through April 2007, BU developed in 79 persons (48 residents and 31 visitors). Initially, all patients were local residents, but in 2004 the outbreak increased in intensity and began to include visitors as well as residents (Figure 4). All case-patients who could be accurately located either lived in or visited Point Lonsdale and the western edge of Queenscliff, and none were linked solely to the main township of Queenscliff.

**Figure 5:**
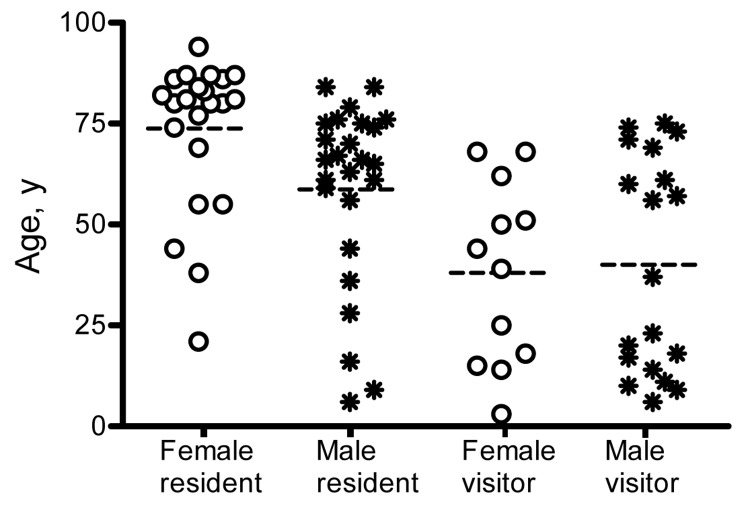
Cases of Buruli ulcer epidemiologically linked to Point Lonsdale, Australia, by resident/visitor status, age, and sex. Dashed lines are medians.

**Figure 6:**
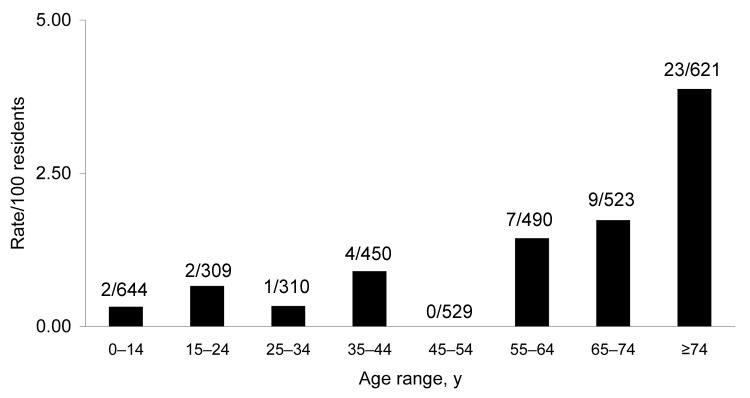
Estimated age-specific attack rates of Buruli ulcer for residents of Point Lonsdale/Queenscliff, Australia (postcode 3225). Values above the bars are cases per total no. residents in each age group.

Most case-patients were adults and many were elderly (Figure 5), although 14 of the 79 were children <18 years of age. Among visitors, there was a bimodal age distribution, with relatively low numbers of adults 20–50 years of age. An estimate of the age-specific attack rate for residents of Point Lonsdale/Queenscliff was obtained with reference to the 2001 Australian census. Because census data were not available for the 2 towns separately, the calculation assumes that the age distribution of Point Lonsdale and Queenscliff is similar. A similar analysis for visitors was not performed because appropriate denominators could not be determined. The risk appeared to increase strongly with age and was ≈7× higher for those >55 years of age than in those <55 years of age (p<0.001) (Figure 6).

The incubation period for residents and for most visitors could not be assessed because exposure to the BU-endemic area occurred repeatedly over a prolonged period. However, in 2006, one patient reported a 1-week contact history with Point Lonsdale (P.D.R. Johnson, unpub. data). Her case was diagnosed and reported 7 months after this exposure.

### Mosquito Testing

A total of 23,692 mosquitoes were captured in Point Lonsdale during a 25-month period; 96% were *Aedes camptorhynchus* (Thomson). Twelve other species comprised the remaining 4% (Table 1). Of 11,504 mosquitoes tested, 48 pools were positive for IS*2404*; of these, 13 pools were also positive for KR and IS*2606*. Forty-one of 48 pools were female *Ae*. *camptorhynchus*, 4 were positive pools of *Coquillettidia linealis* (Skuse), and 1 each were *Anopheles annulipes* (Walker s.l.), *Culex australicus* (Dobrotworsky and Drummond), and *Ae*. *notoscriptus* (Skuse). For 2 positive pools with particularly high *M*. *ulcerans* DNA concentrations, VNTR locus 9 was amplified and the sequence was identical to that of the local outbreak strain cultured from case-patients (*23*).

**Table 1:** Maximum likelihood estimate (MLE) per 1,000 mosquitoes trapped in Point Lonsdale, Australia, and tested by PCR for insertion sequence IS*2404* of *Mycobacterium ulcerans*

Species	No. mosquitoes	No. positive pools/no. pools tested	MLE*	95% Confidence interval
*Aedes camptorhynchus*	10,558	41/757	3.98	2.90–5.35
*Coquillettidia linealis*	480	4/57	8.53	2.80–20.34
*Ae*. *notoscriptus*	221	1/49	4.47	0.26–21.37
*Culex australicus*	76	1/27	12.78	0.77–59.74
*Ochlerotatus alboannulatus*	52	0/16	0	0.00–58.19
*Anopheles annulipes s*. *l*.	49	1/18	22.44	1.24–115.30
*Cx*. *globocoxitus*	43	0/20	0	0–76.19
*Cx*. *annulirostris*	10	0/2	0	0–161.60
*Cx*. *molestus*	6	0/6	0	0–390.33
*Cx*. *quinquefasciatus*	5	0/2	0	0–319.26
*Oc*. *australis*	3	0/2	0	0–499.14
*Cx*. *pipiens gp*.	1	0/1	0	0–793.45
Total	11,504	48/957	4.28	3.20–5.62

*MLE bias was corrected when >1 pool was positive; otherwise uncorrected.

Thirty-five IS*2404*-positive pools did not contain IS*2606* and KR. However, the cycle threshold (Ct) values for IS*2404* were lower for those pools that did have IS*2606* and KR, which suggested that failure to detect KR and IS*2606* in some pools was caused by low DNA concentration, rather than lack of specificity for *M*. *ulcerans*. This finding is consistent with known differences in copy number per cell of targets used for PCR screening and confirmation (*23*). A total of 124 pools of mosquitoes that were negative for IS*2404* by PCR were screened with probes for KR and IS*2606*. None were positive, which indicated that these 2 loci are consistently linked to IS*2404* and do not occur independently.

The MLE (bias corrected) for all mosquitoes over the entire testing period at Point Lonsdale was 4.3 *M*. *ulcerans* PCR-positive mosquitoes/1,000 tested (95% confidence interval [CI] 3.2–5.6). However, mosquito numbers varied widely between trappings, as did proportions of positive pools. On 1 occasion, only 269 mosquitoes were trapped, but 6 of the pools were positive (December 2005; MLE 22.4, 95% CI 10.3–50.3). Most PCR-positive pools had relatively high Ct values for IS*2404* PCR, which indicated low numbers of contaminating *M*. *ulcerans* cells. With reference to spiking experiments under laboratory conditions, ≈10–100 *M*. *ulcerans* were likely to have been present per contaminated mosquito (*23*).

### Mosquito Numbers, Proportion PCR Positive, and Reporting of BU

Trapping was conducted at Point Lonsdale between December 2004 and January 2007. Mosquito numbers varied during the period, and traps were not set when local reports suggested low mosquito numbers (Appendix Figure). There appeared to be a qualitative relationship between PCR-positive mosquitoes in spring and summer (September–February) and reporting of new cases of human disease in autumn and winter (March–August). The exposure-to-reporting interval is typically longer than the actual incubation period because patients do not always seek medical assistance immediately and doctors do not always diagnose BU when a patient is first seen (*28*).

### Mosquitoes Caught at Other Locations in Victoria

To test that the observed association between *M*. *ulcerans* and mosquitoes only occurs in outbreak areas, we tested 3,385 mosquitoes from several inhabited areas with lower BU endemicity than Point Lonsdale. From October 2005 through January 2007, a total of 2,119 mosquitoes (89% *Ae*. *camptorhynchus*) were trapped in townships on the Bellarine Peninsula where 30 cases of BU have been reported in the past 5 years; 3 pools of *Ae*. *camptorhynchus* were positive by IS*2404* PCR. In January and June 2006, a total of 795 mosquitoes (82% *Ae*. *camptorhynchus)* were trapped in the Bass Coast Shire, which includes Phillip Island, a region that has previously been endemic for *M*. *ulcerans* (*14*) but has only reported 2 cases in the past 5 years. One pool of *Ae*. *notoscriptus* was positive for IS*2404*. From February through April 2006, 471 mosquitoes were captured from inhabited areas in northern and central Victoria where no human cases of *M*. *ulcerans* have been reported. Ten different species were trapped, including 226 *Ae*. *camptorhynchus* (48%), but all pools were negative for IS*2404*. When analyzed together, an association was observed between degree of endemicity and probability of trapping mosquitoes that are positive by PCR for *M. ulcerans* (Table 2), but this association did not show statistical significance (p = 0.07).

**Table 2:** Relationship between cases of Buruli ulcer, mosquitoes tested, and maximum likelihood estimate (MLE) per 1,000 mosquitoes trapped in Victoria, Australia, and tested by PCR for insertion sequence IS*2404* of Mycobacterium ulcerans*

Region	No. cases past 5 y	No. mosquitoes tested (% *Aedes camptorhynchus*)†	No. pools positive	MLE (95% CI)
Point Lonsdale	79	11,504 (91.8)	48	4.2 (3.08–5.53)
Bellarine Peninsula (excluding Point Lonsdale)	30	2,119 (88.7)	3	1.42 (0.37–3.85)
Bass coast Shire including Phillip Island	2	795 (82.1)	1	1.25 (0.07–6.03)
Central and northern Victoria (Mildura, Swan Hill, Moira, Shepparton)	0	471 (48.0)	0	0 (0–7.34)
Total	111	14,889 (89.4)	52	3.57 (2.70–4.64)

*MLE bias was corrected when >1 pool was positive, otherwise uncorrected. CI, confidence interval †p value = 0.07 (χ^2^: 4 × 2 table; pools positive/no. tested).

## Discussion

To our knowledge, the outbreak of BU in Point Lonsdale is the largest ever recorded in Australia and has now caused more than twice as many cases as the well-described outbreak at Phillip Island a decade earlier (*16*,*29*). A striking feature of both outbreaks is their intensely localized nature. We identified 79 cases that were epidemiologically linked to Point Lonsdale and the western edges of Queenscliff, but the town of Queenscliff, only 4 km to the east along the same beach, has so far remained disease free. The cumulative attack rate for both towns is estimated to be 1.2% of the resident population, but it could be up to twice as high if only the population of Point Lonsdale, where all transmission appears to have occurred, were considered. Although Queenscliff remains unaffected, the nearby towns of Barwon Heads and Ocean Grove, ≈12 km west of Point Lonsdale, began reporting their first cases in 2005.

The first case at Point Lonsdale was reported in January 2002. In 2004, the outbreak increased in intensity and began to involve visitors as well as residents, which suggested that environmental contamination with *M*. *ulcerans* has steadily increased over 5 years. Among local residents, we found a higher attack rate in the elderly, with 3.7% of residents of Point Lonsdale/Queenscliff >75 years of age with BU. The reasons for this age distribution are not known, but increasing risk with age could be caused by an age-related immune defect or an unrecognized behavioral factor. Among visitors, there was a pronounced bimodal age distribution, which probably represents a skewing of the exposed population (e.g., young children going to stay with their retired grandparents over the summer while their parents stayed at work) but may also reflect increased susceptibility in young persons. This bimodal pattern, which included increased incidence in young persons and the elderly, has also been reported in Africa (*30*).

During our investigations at Point Lonsdale, we focused initially on several marshy areas and obtained positive PCR results for plant material from 2 small ornamental lakes and soil from storm water drains (*23*). However, case-patients did not report direct contact with these lakes or drains (these sources of water are not used for swimming or wading). Thus, how people were exposed is not clear. In an outbreak in Phillip Island, many cases were clustered around a newly formed wetland and a golf course irrigation system, and we proposed transmission from these sites by aerosol (*16*,*29*). However, this hypothesis may not be supported by our new evidence, which suggests that *M*. *ulcerans* may not be free-living in the environment but may have adapted to specific niches within aquatic environments, including salivary glands of some insects. Thus, we investigated whether *M*. *ulcerans* could be detected in mosquitoes, which had been reported in higher than usual numbers at Point Lonsdale. We also investigated behavior in a case-control study (the subject of a separate report), which found that being bitten by mosquitoes increased the odds of having BU (*31*).

A total of 14,889 mosquitoes obtained over a 25-month period (11,504 from Point Lonsdale) were tested for *M*. *ulcerans* by using a highly sensitive and specific real-time PCR (*23*). We used PCR because direct culture of *M*. *ulcerans* from the environment is extremely difficult and was only achieved when IS*2404* PCR screening of environmental samples accurately directed researchers to specific microenvironments that include water insects and aquatic plants (*32*). Although IS*2404*, IS*2606*, and the mycolactone-producing virulence plasmid have been detected in mycobacteria other than *M*. *ulcerans* (*33*–*35*), identification of these targets in expected relative proportions and the VNTR locus 9 sequence identical to that of the outbreak strain in a subset of mosquito pools with sufficiently high DNA concentrations confirms that we identified the outbreak strain (*23*).

We also demonstrated that over a 2-year cycle at Point Lonsdale absolute numbers of mosquitoes and PCR-positive mosquitoes increased in spring and summer followed by a cluster of new human cases in autumn and winter. This pattern is consistent with recent point estimates that suggest the incubation period for BU in Australia is 3–7 months (2 cases) (*36*) and 1–4 months (3 cases) (*28*), and that an additional 1–6 weeks may elapse before cases are diagnosed and reported (*28*).

The predominant species trapped was *Ae*. *camptorhynchus*; however, identification of *M*. *ulcerans* in 4 other species suggests that *M*. *ulcerans* contamination of mosquitoes is not species specific. *Ae*. *camptorhynchus* is a salt marsh species, an aggressive biter, and a major pest in coastal areas of southeastern Australia that has been linked to transmission of Ross River virus. The mosquito appears in large numbers after rain as minimum temperatures begin to increase, with a lag time of ≈1 month (*37*). Of the other species from which at least 1 PCR-positive pool was identified, *An*. *annulipes* and *Cq*. *linealis* are fresh water species (*38*). *Ae*. *notoscriptus* is a peridomestic species that breeds in containers (e.g., in roof gutters) (*39*), can transmit dog hookworm, and has a limited flight range (e.g., <200 m) (*40*). In contrast, *Cx*. *australicus* may have a flight range of many kilometers (*41*). A limited number of other biting or aquatic insects were also tested and none were positive for *M*. *ulcerans*. However, larger numbers must be screened before it can be concluded that they do not transmit *M*. *ulcerans*.

Our results do not demonstrate viability or transmissibility of *M*. *ulcerans* at the time mosquitoes were captured, and the method we used does not answer questions about location of *M*. *ulcerans* within the insect. Because *M*. *ulcerans* is an environmental pathogen, PCR-positive mosquitoes may only be indicators of its presence in the environment and not linked to transmission. The Ct values obtained for mosquito pools suggest that only 10–100 organisms were present per positive pool, which is more consistent with organisms being acquired on outer surfaces of mosquitoes when resting or feeding in storm water drains (*23*), rather than mosquitoes being a true productive reservoir and vector. However, if some bacterial cells were present on the proboscis, they could have been injected beneath the keratin layer during feeding. Although the inoculum size required to cause a human infection is unknown, the long incubation period suggests a low initial inoculum. Our findings do not demonstrate that mosquitoes are responsible for transmission, but this possibility should be investigated. Studies are underway to artificially infect mosquito larvae with *M*. *ulcerans* and initiate infection in a mouse model, as has been conducted with naucorids (*9*).

Although our findings may not apply to the situation in Africa, the close genetic relationship of Australian isolates of *M*. *ulcerans* with strains from humans with BU in Africa (*35*) should encourage similar search on *M*. *ulcerans* in mosquitoes from the primary BU-endemic regions of West Africa. We have shown that a small proportion of mosquitoes of 5 species captured in a BU-endemic area during an intense human outbreak of BU can carry *M*. *ulcerans*; PCR-positive mosquitoes are likely present at times of peak transmission and mosquitoes captured in areas with few human cases appear less likely to be positive for *M*. *ulcerans*. We hypothesize that transmission by mosquitoes offers a partial explanation for the outbreak at Point Lonsdale and possibly at other sites in southeastern Australia.

## Supplementary Material

Appendix Figure Relationship between reporting of cases of Buruli ulcer (BU) and mosquitoes tested from Point Lonsdale, Australia, December 2004-January 2007. Increased mosquito activity in spring and summer (September-February) appears to be followed by a wave of new reports in autumn and winter (March-August). A) No. mosquitoes tested by month at Point Lonsdale (traps were not set when local reports suggested low mosquito activity). B) Proportion of tested mosquitoes positive by PCR for Mycobacterium ulcerans by month. C) No. of new cases of BU epidemiologically linked to Point Lonsdale by month.
