# Supplementary figures and images for: Mycobacterium ulcerans in Mosquitoes Captured during Outbreak of Buruli Ulcer, Southeastern Australia
Source: Emerg Infect Dis. 2007 Nov;13(11):1653–60. doi: 10.3201/eid1311.061369 (PMC3375796; doi:10.3201/eid1311.061369)

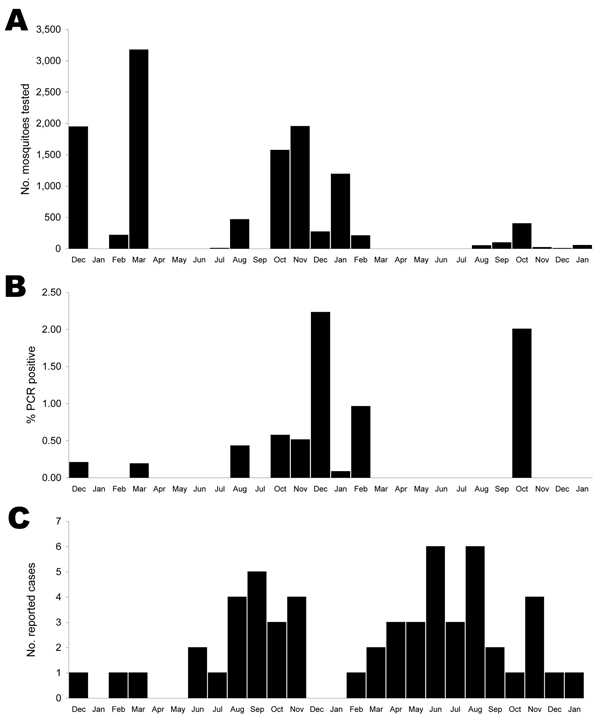

Supplement: Appendix Figure — Relationship between reporting of cases of Buruli ulcer (BU) and mosquitoes tested from Point Lonsdale, Australia, December 2004-January 2007. Increased mosquito activity in spring and summer (September-February) appears to be followed by a wave of new reports in autumn and winter (March-August). A) No. mosquitoes tested by month at Point Lonsdale (traps were not set when local reports suggested low mosquito activity). B) Proportion of tested mosquitoes positive by PCR for Mycobacterium ulcerans by month. C) No. of new cases of BU epidemiologically linked to Point Lonsdale by month. [file 06-1369_appF-s1.gif]
